# Informal Caregivers’ Needs in Post-Stroke Home Care: Insights from Focus Groups Conducted in Portugal Within the CaregIVR Project

**DOI:** 10.3390/nursrep16090311

**Published:** 2026-09-01

**Authors:** Helena José, Martina Giusti, Lara Costa e Silva, Diogo João Tomás, Susana Ganhão-Arranhado, Carla Silva, Luís Manuel Mota de Sousa

**Affiliations:** 1 Nursing Department, Atlântica School of Health, 2730-036 Barcarena, Portugal; 2 Health Sciences Research Unit: Nursing, Coimbra Nursing School, 3004-011 Coimbra, Portugal; 3 Nutrition Department, Atlântica University, 2730-036 Barcarena, Portugal; 4 Engineer Department, Atlântica University, 2730-036 Barcarena, Portugal; 5 Comprehensive Health Research Centre (CHRC), Universidade de Évora, 7000-671 Évora, Portugal

**Keywords:** caregivers, stroke, home care, immersive virtual reality, health literacy, qualitative research

## Abstract

**Background/Objectives:** This study aimed to identify and categorise the educational, training, and support needs of informal caregivers of stroke survivors in order to inform the development of an Immersive Virtual Reality (IVR) intervention within the European caregIVR project (Project No. 101129454; EU4Health Programme). **Methods:** A qualitative descriptive study was conducted using three focus groups involving informal caregivers, healthcare professionals and other stakeholders, and academics and healthcare students in Portugal (n = 33). Discussions explored everyday caregiving challenges, priority educational needs, and expectations regarding digital support tools. Data were analysed using qualitative content analysis with an inductive–deductive coding strategy supported by NVivo software (version 14). **Results:** Participants identified the transition from hospital to home as the most critical stage of the caregiving journey, characterised by poor continuity of care and insufficient preparation before hospital discharge. Major challenges included limited stroke-related health literacy, difficulties adapting the home environment, and substantial emotional, financial, and social burdens that frequently resulted in social isolation and caregiver burnout. Participants regarded IVR as a promising educational strategy for delivering practical, repetitive, and low-risk skills training. **Conclusions:** The findings suggest that caregiver empowerment should be promoted through progressive, person-centred, and non-judgemental educational programmes tailored to caregivers’ evolving needs. Caregivers should be supported by multidisciplinary teams capable of addressing the clinical, practical, and psychosocial complexity of post-stroke home care. Rather than replacing professional support, IVR should be considered a complementary educational tool with the potential to improve caregivers’ preparedness, confidence, and safety in the home-care setting.

## 1. Introduction

Cardiovascular diseases (CVDs) remain one of the leading causes of morbidity and mortality worldwide, requiring a progressive shift from acute hospital-based treatment to long-term rehabilitation and community care [1]. Within this continuum of care, informal caregivers play a pivotal role by bridging the transition between hospital discharge and home-based rehabilitation. An informal caregiver is often a family member who provides care, typically unpaid, to someone with whom they have a personal relationship [2]. Their contribution is particularly crucial following stroke, where the sudden onset of physical, cognitive, and behavioural impairments places substantial clinical and practical demands on caregivers from the moment of discharge [3]. Stroke remains the second leading cause of death worldwide and the third leading cause of death and disability combined, measured as disability-adjusted life years (DALYs). Approximately 12 million new strokes occur annually, while nearly 94 million people worldwide are currently living with the consequences of stroke. Between 1990 and 2021, the global burden of stroke increased substantially, with marked rises in incidence, prevalence, and mortality, disproportionately affecting low- and middle-income countries [4,5].

The transition to home-based care frequently imposes a substantial and multifaceted burden on the caregiver [6]. The burden associated with home-based stroke care is multidimensional and influenced by several factors, including the patient’s physical and cognitive dependency, caregivers’ own health status, and the availability of social, family, and financial resources [7,8].

Caregivers often report high levels of psychological distress, including anxiety and depression, alongside a decline in their own physical health [9,10,11]. The demands of constant supervision often lead to social frailty due to their isolation [12,13] and significant financial pressure, stemming from both out-of-pocket medical expenses and a reduction in professional productivity, often leading to burnout [14,15].

Critically, when a caregiver is ill-prepared, the consequences extend beyond their personal well-being, potentially compromising the quality of care and increasing the risk of adverse outcomes and hospital readmissions for the patient [16,17].

Despite this growing body of evidence, structured interventions specifically designed to prepare caregivers for the transition to home remain limited [18,19]. Effective support should move beyond the passive delivery of information and promote the acquisition of practical skills through active learning strategies. In this context, digital health technologies—and particularly Immersive Virtual Reality (IVR)—have emerged as promising educational tools capable of providing scalable, safe, and realistic learning environments that bridge the gap between hospital discharge and home care [20,21].

Although previous studies have evaluated educational interventions aimed at improving caregivers’ ability to respond to care needs, relatively little attention has been paid to proactive, experiential, and sustainable training approaches capable of strengthening caregivers’ confidence and preparedness over time. Such approaches have the potential to transform caregiving from a reactive response to everyday challenges into a proactive and informed process.

Recent evidence—such as the work of Brydon et al. (2021)—suggests that IVR may enhance caregivers’ empathy by enabling them to experience patients’ sensory and cognitive impairments firsthand [22]. Furthermore, as highlighted by Hirt & Beer (2020) [23], immersive simulation has been associated with reduced stress and anxiety, while providing a safe environment in which caregivers can acquire practical skills before applying them in real-life settings [23,24]. However, there is a notable gap in evidence-informed interventions specifically tailored for informal caregivers of post-stroke patients.

The present study aimed to identify and categorise the educational and support needs of informal caregivers providing home care to stroke survivors in Portugal, with the ultimate goal of informing the development of IVR-based educational interventions.

The study forms part of the European caregIVR project (“Cardiovascular Health Promotion: The Impact of Immersive Virtual Reality”; Project No. 101129454; EU4Health Programme), which seeks to improve cardiovascular health literacy and empower informal caregivers through innovative digital educational strategies. Within Work Package 3 (“Development of Good Transnational Practices”), Action 3.1 (“Focus Groups on the App and IVR Design”), focus groups were conducted across participating countries to identify caregivers’ priorities and guide the design of immersive training scenarios. The present paper reports the findings from the Portuguese focus groups.

## 2. Materials and Methods

### 2.1. Study Design

This study adopted a qualitative descriptive design using focus groups to explore the educational and support needs of informal caregivers of stroke survivors, following Sandelowski’s (2010) methodological framework [25]. The study followed the Consolidated Criteria for Reporting Qualitative Research (COREQ) to ensure transparency and methodological rigour [26]. The study aimed to identify and categorize informal caregivers’ needs in providing home care for stroke survivors in Portugal. This methodological approach is particularly suitable when the goal is to provide a comprehensive description of participants’ experiences and needs while remaining close to the data, maintaining a low level of interpretation, known as “low inference” [25].

A literature review was conducted to identify the main thematic areas related to the educational and training needs of informal caregivers, with particular attention to CVD prevention, post-stroke home care, and health promotion. The findings informed the development of the semi-structured focus group guide used in the second phase of the study (Appendix A).

This study was grounded in the interpretivist paradigm, which assumes that reality is socially constructed and can be understood through individuals’ subjective interpretations of their lived experiences [26].

Within this framework, the focus groups were designed to generate an in-depth understanding of informal caregivers’ educational and support needs, thereby informing the development of training interventions tailored to their actual knowledge gaps and everyday caregiving experiences [16].

### 2.2. Participants and Eligibility Criteria

Informal caregivers’ educational and training needs were explored through face-to-face focus groups [27,28]. Purposive sampling was adopted, following the recommendations of Krueger and Casey (2014) [29] to recruit participants with direct experience and complementary perspectives on post-stroke caregiving. This approach enabled the exploration of the phenomenon from different viewpoints while ensuring that all participants shared relevant knowledge and experience of the topic under investigation.

Participants were purposively recruited from three predefined groups:

- Informal caregivers of post-stroke patients, the primary target population of the training sessions.
  Inclusion criteria: current or previous experience providing home care to a stroke survivor.
  Exclusion criteria: caregiving experience limited to individuals with conditions other than stroke. Caregivers with different levels of caregiving experience, patient dependency, and time since stroke were intentionally included to ensure a broad representation of educational needs and caregiving experiences, consistent with the aims of the study.
- Health professionals and other stakeholders (e.g., patient representatives, IT companies, organizations involved in post-stroke care), selected because of their role in caregiver education, support services and the development of innovative care solutions.
  Inclusion criteria: demonstrated experience in the design, implementation, delivery, or evaluation of educational programmes or support initiatives for informal caregivers of post-stroke patients across the continuum of care, including acute care, transitional care (e.g., protected discharge services), and home care. Stakeholders not directly involved in training activities (e.g., IT companies and patient organizations) were required to demonstrate a relevant contribution to caregiver support, education, or the development of digital health solutions for post-stroke care.
  Exclusion criteria: professional experience limited to the clinical management of post-stroke patients without involvement in caregiver training, education, or support activities.
- Academics and healthcare students, included because of their current or potential role in the education and training of informal caregivers. Inclusion criteria: academics with demonstrated expertise in teaching or curriculum development related to caregiver education or post-stroke care; healthcare students enrolled in a health-related degree programme who had completed clinical placements or educational projects involving post-stroke care or caregiver education.
  Exclusion criteria: academics or students whose experience was exclusively related to the clinical care of post-stroke patients without involvement in education or caregiver training.

Including these three participant groups enabled data triangulation by incorporating complementary experiential, professional, educational, and organizational perspectives on informal caregiving after stroke.

Potential participants were identified through caregiver associations, healthcare organisations, patient organizations, project partners, and universities involved in the study. Invitations to participate in the face-to-face focus groups were distributed via email by the principal investigator (H.J.) through the collaborating organizations. The invitation included an overview of the caregIVR project, the aims of the study, and information regarding participation in the focus groups. Because recruitment was mediated through partner organizations, it was not possible to determine the number of individuals who declined participation or to calculate response and dropout rates. Nevertheless, all participating organizations were represented by at least one participant in the focus groups [30].

The informal caregivers who participated in the focus groups had previously been involved in other activities of the European caregIVR project, within which the present study was conducted. Specifically, they had completed a questionnaire assessing caregiver burden among informal caregivers of people living with stroke. Their previous participation facilitated their familiarity with the project’s objectives but did not influence the focus group discussions, which explored caregivers’ educational and support needs through open-ended questioning.

### 2.3. Data Collection

Data were collected through three face-to-face focus groups conducted between March and June 2024. The focus groups were held at the premises of local caregiver associations and partner organizations to ensure an accessible and familiar environment, particularly for informal caregivers, who represented the study’s primary and potentially more vulnerable participant group [29].

Before each focus group, participants received information about the aim and procedures of the study and were invited to provide informed consent. All participants signed an informed consent form for participation in the caregIVR project and a separate consent form authorizing the processing of personal data in compliance with the European Regulation (EU) 2016/679 (GDPR).

No individuals other than the participants and the research team were present during the focus groups, thereby minimizing external influences on the discussions and ensuring confidentiality.

An interview guide was developed based on the objectives of the caregIVR project, the aims of the present study, and the relevant literature. The guide consisted of open-ended questions and prompts designed to facilitate discussion while allowing participants to introduce issues they considered relevant. The interview guide was used by the facilitators throughout the data collection process and was not shared with participants in advance.

Each focus group was moderated by one experienced facilitator, while one or two additional researchers acted as non-participant observers, taking field notes on group dynamics, non-verbal communication, and contextual factors that complemented the audio recordings.

The composition of the research team for each focus group was as follows:

Focus Group 1—Informal caregivers. Facilitator: H.J. Observer: M.G. Focus Group 2—Health Professionals and other stakeholders. Facilitator: S.G.-A. Observer: L.C.e.S. Focus Group 3—Academics and healthcare students. Facilitator: L.M.M.d.S. Observers: D.J.T. and C.S.

Before each session, the facilitators and observers introduced themselves as members of the caregIVR research team and explained the objectives of the project, with particular emphasis on the purpose of the focus group discussion.

All researchers involved in data collection were academics or professional researchers with experience in qualitative health research. With the exception of one researcher who was completing doctoral studies at the time of the study, all held a PhD. The multidisciplinary research team included both female and male researchers with expertise in qualitative methodologies, health sciences, and multicultural approaches to healthcare. This diversity was intended to promote reflexivity and reduce potential disciplinary, cultural, and gender-related influences on data collection and interpretation.

Each focus group lasted approximately 90–120 min and was conducted once. All discussions were audio-recorded with participants’ consent and subsequently transcribed verbatim. Approximately one week after each focus group, the transcripts were returned to participants for member checking to verify their accuracy and provide an opportunity to clarify, modify, or expand their contributions. No participants requested changes or provided additional comments.

### 2.4. Data Treatment and Analysis

Audio recordings from the three focus groups were reviewed by the research team before being transcribed verbatim by one investigator [31]. Following transcription, two researchers independently assessed the transcript for completeness, accuracy, and richness of data [25]. Redundant statements within each focus group were removed while preserving the specificity of each discussion. Data saturation was supported by the consistency of participants’ accounts and the recurrence of themes across three focus groups.

The transcripts were subsequently subjected to qualitative content analysis using an inductive–deductive coding approach [32,33], supported by NVivo software (version 14). The analytical process comprised four stages:

i. Definition of the study objectives and analytical framework;
ii. Construction of the analytical corpus;
iii. Development and refinement of categories according to the criteria of homogeneity, relevance, representativeness, and exhaustiveness;
iv. Identification of meaningful units of analysis.

Initially, one researcher conducted open coding by identifying meaningful text segments and assigning preliminary codes (H.J.). The coding framework was informed by the literature and by repeated readings of the transcripts, allowing both predetermined and data-driven categories to emerge. Similar codes were progressively grouped into broader categories, which were refined through an iterative process of constant comparison across transcripts. This process led to the identification of four overarching categories that were consistently represented across all focus groups.

Throughout the analysis, two experienced qualitative researchers independently reviewed the coding framework and compared their interpretations with those of the primary coder. Any discrepancies were discussed during regular meetings with the research team until consensus was reached. The researchers also engaged in continuous reflexive discussions to critically examine how their disciplinary backgrounds, assumptions, and prior experiences might influence data interpretation. This iterative and reflexive analytical process ensured that the final categories remained grounded in participants’ accounts while accurately reflecting the meanings co-constructed during the focus group discussions [31,33]. Participants did not provide feedback on findings.

### 2.5. Study Rigor

Methodological rigor was ensured throughout the study in accordance with the criteria of credibility, dependability, confirmability, and transferability. Credibility was enhanced through investigator triangulation, whereby two experienced qualitative researchers (S.G.A. and L.M.M.d.S.) independently reviewed the coding framework and discussed emerging interpretations with the wider research team (M.G.; L.C.e.S.; D.J.T. and C.S.) until consensus was achieved. Member checking was also undertaken by inviting participants to review the interview transcripts and the preliminary interpretation of the findings, thereby confirming that the results accurately reflected their experiences. Dependability was strengthened through the application of a systematic and transparent analytical process, including the use of an explicit coding framework, iterative category refinement, and regular research team meetings to ensure consistency in coding decisions and theme development.

Confirmability was promoted through ongoing reflexive discussions among the researchers and by supporting the findings with verbatim participant quotations, enabling readers to assess the relationship between the data and the resulting interpretations. Finally, transferability was facilitated by providing a detailed description of the study context, participant characteristics, data collection procedures, and analytical process, allowing readers to evaluate the applicability of the findings to other contexts and populations.

### 2.6. Ethical Considerations

This study was conducted as part of the European caregIVR project, which received ethical approval from the Ethics Committee of the Atlântica School of Health (approval date: 26 January 2024, code CE01_2024).

Before participation, all individuals received written and verbal information about the aims of the study, the focus group procedures, the voluntary nature of participation, their right to withdraw at any time without consequences, and the measures adopted to ensure confidentiality. Written informed consent was obtained from all participants prior to data collection.

To protect participants’ privacy, all data were anonymized by assigning a unique identification code to each participant (e.g., P1, P2, P3). Audio recordings and verbatim transcripts were de-identified by removing any personally identifiable information before analysis. Only the anonymized transcripts were imported into the qualitative data analysis software.

All study data were managed in accordance with the principles of the Declaration of Helsinki and the European General Data Protection Regulation (Regulation (EU) 2016/679, GDPR). Electronic files were stored on a secure, password-protected institutional cloud platform accessible only to authorized members of the research team. The final research dataset will be retained in encrypted form, with access restricted to the principal investigator, in accordance with institutional data management policies.

## 3. Results

This paper reports the qualitative findings from focus groups conducted in Portugal, aiming to capture the lived experiences and specific needs of caregivers to ensure that the digital solutions developed are both culturally sensitive and clinically relevant.

### 3.1. Participant Overview

A total of 33 people participated in the meetings, representing various stakeholder groups: 9 informal caregivers, 11 health professionals and other stakeholders, and 13 academics and healthcare students. Within the sample, 85% were female and 15% male (Table 1). Average age was 43.3 ± 11.8 years (Min: 21; Max: 66). The age structure of the sample was diverse: 3 participants were aged 18–24, 5 were aged 25–34, 8 were aged 35–44, 10 were aged 45–54, 6 were aged 55–64, and, at the end, 1 was over 65.

### 3.2. Qualitative Findings

The reported coding tree presents a hierarchical overview of the main needs of post-stroke caregivers identified through the qualitative analysis.

**Figure 1:**
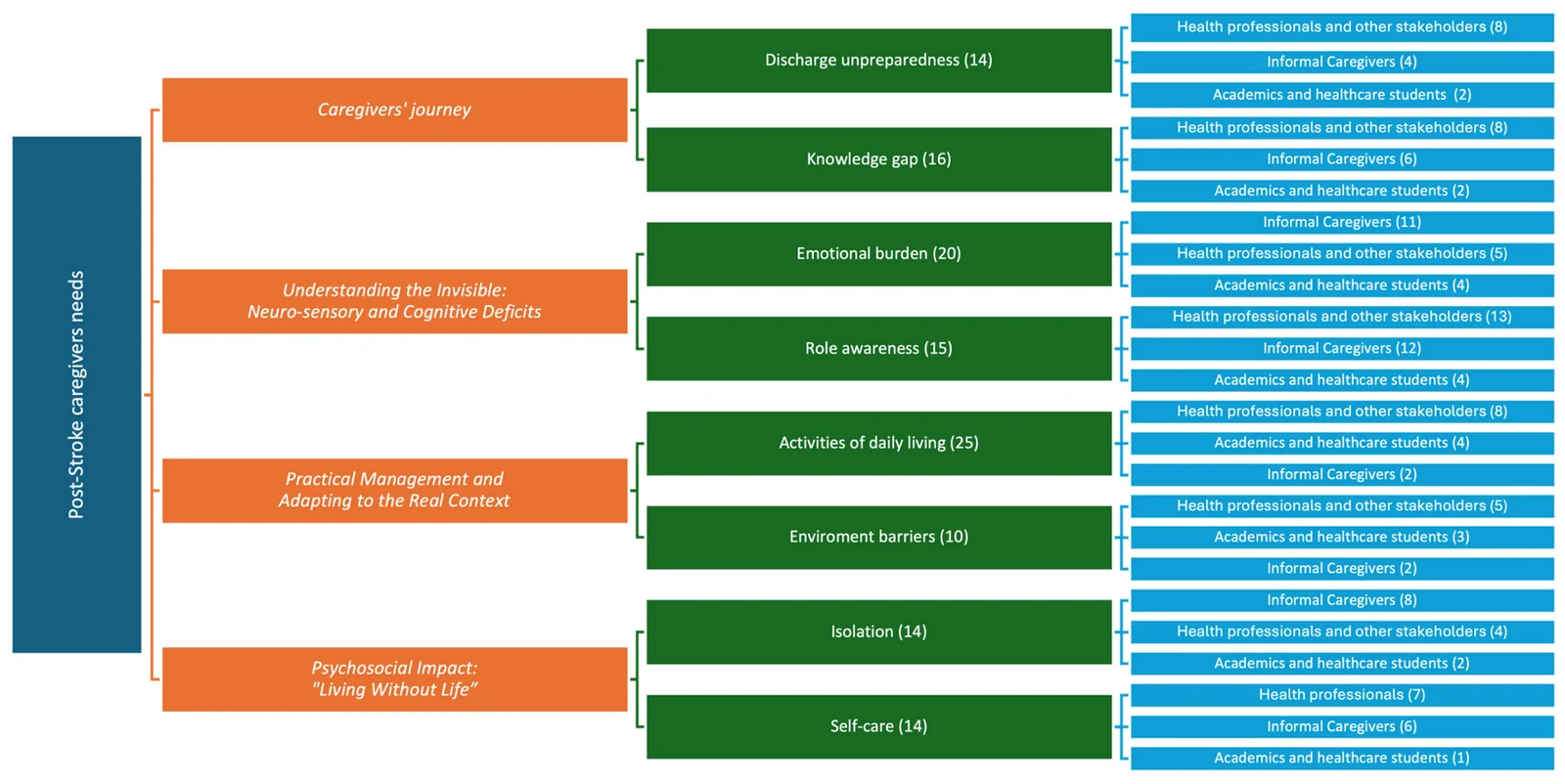
Conceptual scheme of qualitative findings on post-stroke caregivers’ needs. Source: Authors’ own elaboration based on focus group data (2024). Note: Numbers in brackets represent the frequency of references to each category across the three focus groups (illustrative purposes only, not quantitative analysis).

Qualitative content analysis identified four overarching themes describing caregivers’ educational and support needs during the transition from hospital to home, reported in the coding tree (Figure 1). Although conceptually distinct, these themes were closely interconnected and collectively reflected the complexity of the caregiving experience.

- Caregivers’ Journey, which includes discharge unpreparedness (no. 14) and knowledge gap (no. 16), highlighting the challenges caregivers face during the transition from hospital to home care.
- Understanding the Invisible: neuro-sensory and cognitive deficits, which encompass emotional burden (no. 20) and role awareness (no. 15), emphasizing the psychological impact of caregiving and the need to better understand often invisible cognitive and sensory impairments.
- Practical Management and Adapting to the Real Context, which includes activities of daily living (no. 25) and environmental barriers (no. 10), reflecting the practical challenges associated with managing daily care and adapting the home environment.
- Psychosocial Impact: “Living Without Life”, which comprises isolation (no. 14) and self-care (no. 14), illustrating the social and personal consequences of long-term caregiving.

These four categories are presented separately for simplicity of representation but, in reality, they are strongly interconnected.

The right-hand column further illustrates how each theme was reported across the different stakeholder groups (informal caregivers, healthcare professionals and other stakeholders, and academics and healthcare students), providing an overview of the distribution of perspectives within the sample.

Due to the consistency between data obtained in the different focus groups, participants remarked on the same four significant presented categories and the related themes.

#### 3.2.1. Caregiver’s Journey

Both caregivers and healthcare professionals described hospital discharge as a critical and destabilizing phase in which everyday life becomes dysfunctional. This situation was mainly attributed to discontinuities in care, insufficient coordination between services, and inadequate communication and information provided to caregivers.

Health professionals particularly highlighted a lack of coordination between hospital discharge processes and community-based support services. *“Lack of articulation that exists between leaving the hospital and coming home. Because, many times, they leave the hospital, come home and say that now I am going to the family doctor and deliver this letter.(…) Then they come to the family doctor and then they need to make an appointment. And then they need to go to the appointment”* (FG2 P5). This fragmentation often contributes to the deterioration of patients’ health conditions, escalation into emergency situations, and subsequent rehospitalizations. Professionals also highlighted how these gaps in continuity of care substantially increase the caregiving burden placed on family members during the post-discharge period. Also emphasized the significant increase in the caregiving burden placed on family members during this transition period: *“There was a change in the family and an exchange of roles. I went from being a daughter to a mother and, together with my father, we looked after my mum and I continued to look after her. Nowadays, it’s just me”* (FG1 P4).

Within this context, caregivers emphasised feeling overwhelmed, unsupported, and unprepared to provide appropriate care at home. A major source of difficulty concerned the communication style adopted by healthcare professionals. Many caregivers described struggling to understand complex medical terminology, creating confusion about both the patient’s clinical condition and the practical management of care needs: *“As far as the caregivers is concerned, it’s about feeling more secure in providing care, yes, feeling comfortable and knowing that sometimes what we need is to know that we’re doing well”* (FG1 P2). Caregivers therefore expressed a strong need for communication that is clearer, simpler, and more accessible to properly address patients’ health needs, the expected caregiving tasks, and the community resources available to support them: *“There is also a communication barrier here and an emotional lability with the underlying pathology that makes this assessment difficult. I can’t guarantee that I’m providing the necessary emotional support because I have no way of reliably assessing it”* (FG1 P6).

These findings underline the urgent need for caregiver-oriented education and support programs. Participants highlighted the importance of receiving practical guidance not only on recognizing early warning signs of health deterioration and managing everyday caregiving activities, but also on navigating broader legal, administrative, and community-related issues.

Caregivers expressed the need for support regarding legal rights, inheritance matters, property management, and bureaucratic procedures, all of which became additional sources of stress during the caregiving journey: *“I also often need legal support, particularly because there are questions that arise in relation to the elderly, such as inheritances, sales that family members and children sometimes want to make, how this relates to the elderly or even in terms of the rules of how institutions operate, what they can do, what they can’t do and what rights we have as responsible family members—sometimes I also have doubts and I don’t know where to turn”* (FG1 P4).

While professionals primarily focus on knowledge gaps related to clinical safety, such as recognizing warning signs and performing appropriate care procedures, caregivers are more affected by gaps in navigating healthcare and social support systems, including understanding their rights, managing bureaucratic processes, and coping with the uncertainty and unpredictability of the disease trajectory.

#### 3.2.2. Understanding the Invisible: Neuro-Sensory and Cognitive Deficits

A major theme emerging from the discussions was the difficulty caregivers experience in understanding non-physical and often untouchable impairments. Caregivers described significant challenges in interpreting cognitive, behavioural, and perceptual limitations, which frequently led to misunderstandings of the cared person’s behaviour and intentions. Many participants expressed frustration when faced with apparently contradictory reactions, as illustrated by one caregiver who explained: “*I tell her something to do and her brain seems to. She hears one thing, but her brain does another*” (FG1 P5). Several caregivers described a strong desire to better understand the lived experience of the condition itself, expressing the wish to see the world from the patient’s point of view. One participant stated: “*I’d like to know what’s happening in her case… eventually seeing the world through her eyes and being able to experience this mental confusion*” (FG1 P7).

Experts further emphasized that neurological and cognitive impairments—such as apraxia—are frequently misunderstood and mistakenly interpreted as unwillingness, “*laziness*,” or “*stubbornness*.” As one professional explained: “*Why can’t he comb his hair? Is he being difficult? Is he lazy?… Sometimes it is difficult to explain these issues*” (FG2 P2). Such misinterpretations may negatively affect both the caregiving relationship and the quality of support provided. To address these challenges, academics proposed the use of Immersive Virtual Reality (IVR) as an innovative educational and empathy-building tool for caregivers. Through immersive simulations, caregivers could directly experience some of the difficulties faced by patients, fostering greater understanding and emotional engagement. Suggested examples included watching videos in unfamiliar languages to simulate aphasia or navigating environments with visual field impairments, such as hemianopsia, in order to reproduce the confusion and disorientation experienced by affected individuals.

#### 3.2.3. Practical Management and Adapting to the Real Context

As mentioned before, the transition from hospital to home was consistently described as a critical turning point in which daily life becomes dysfunctional. Caregivers portrayed the return home as a disruptive and emotionally overwhelming experience, characterized by uncertainty, fear, and the need to rapidly adapt to new practical and relational challenges.

A recurring concern among caregivers was the fear of inaccessibility, understood not only in physical terms but also as a psychological and emotional barrier. Participants expressed anxiety about their ability to adequately manage the cared person’s needs within the home environment and to cope with the complexity of new caregiving responsibilities. Among the practical challenges, dysphagia emerged as one of the main sources of anxiety. Caregivers described swallowing difficulties as particularly distressing and difficult to manage, with one participant stating: “*Her swallowing system, the swallowing part, is very, very messed up… it’s all a mess*” (FG 1 P2). Healthcare professionals confirmed that the fear of choking represents a major concern that affects not only caregivers but the entire family unit, often generating constant vigilance and emotional distress during meals and daily care activities.

The home environment itself was frequently perceived as inadequate or unprepared to support the patient’s return. Professionals emphasized that, as discharge approaches, urgent practical questions arise regarding the suitability of the living space: “*What conditions does the house have to receive the person? The bed? The bathroom? The doors? The carpets?*” (FG2 P6). These concerns underline the importance of preparing caregivers not only theoretically, but also through practical and experiential training tailored to real-life domestic contexts. In this regard, participants highlighted the potential value of IVR training scenarios that could allow caregivers to rehearse everyday caregiving activities in a safe and controlled environment: “*I think that, above all, the scenario 3D has to be a home with all its rooms. In fact, in every division there is a challenge: personal hygiene in the bathroom, the transfer from wheelchair to bed in the bedroom, the techniques against choking or the strategies for good nutrition and hydration in the kitchen, generally all the technical aids that exist, and all the panoply that is for specific cases. If you can visualize in 3D how to do it, it’s much easier. That’s what I wanted to ask*” (FG3 P11). Suggested applications included practicing sequential and physically demanding tasks, such as transferring a patient from a wheelchair to a car, navigating domestic spaces safely, or managing emergency situations. Such immersive simulations could improve both practical competence and caregiver confidence before the patient’s return home. In addition, some caregivers recognised feelings of discomfort or shame when performing intimate personal care activities, particularly hygiene-related tasks. These experiences point to the need for training programs that address not only technical caregiving skills, but also the emotional and psychological dimensions of caregiving, helping individuals develop confidence, emotional preparedness, and coping strategies for managing sensitive aspects of care.

#### 3.2.4. Psychosocial Impact: “Living Without Life”

Caregivers described a profound emotional burden associated with caregiving, often characterised by a progressive loss of personal identity and the perception of living a life centred entirely on the needs of another person: “*Unfortunately, all areas of my life have been affected, as I now have more time to look after my mum than to live, in other words, (…) I’ve taken over from my mum, the roles have been swapped on an emotional level and I’m in permanent mourning*.” (FG1 P3). Caregivers described their daily existence as a condition of permanent mourning, characterized by the loss of personal identity, future expectations, and emotional well-being. Several testimonies highlighted the extreme severity of this psychological distress. One participant, in particular, stated: “*I’ve completely cancelled my dreams… I’ve attempted suicide three times because I simply wanted to end the pain… it’s tiredness*” (FG1 P8). These accounts underline how caregiving can evolve into an all-consuming experience, marked by chronic exhaustion, emotional suffering, and feelings of hopelessness.

Within this context of profound emotional strain, caregivers expressed the need for more than practical instructions or technical guidance. Many participants emphasized the importance of receiving recognition, reassurance, and validation for their caregiving efforts. As one caregiver explained: “*We need people to validate our performance and that these people have knowledge of what they’re assessing, because sometimes it’s not easy to find someone who lives up to our expectations and who can advise us*” (FG1 P2). This highlights the caregivers’ desire to feel acknowledged, understood, and supported by competent professionals who can recognize both the complexity of caregiving and the emotional investment it requires.

Healthcare professionals further stressed that caregiver well-being should be considered a fundamental prerequisite for sustainable care. In particular, they emphasized that “*to be a good caregiver, you must take care of yourself first*” (FG2 P11), highlighting the importance of promoting self-care strategies in order to prevent physical exhaustion, psychological burnout, and emotional collapse. This perspective reinforces the need for interventions that support not only patients, but also the mental and emotional health of caregivers themselves.

At the same time, participants identified social isolation as a major factor exacerbating caregiver distress. Many caregivers described feeling alone, disconnected, and excluded from social life because of their caregiving responsibilities. As one health professional stated: “*Caregivers are very isolated… there should be a way of meeting on the European project’ platform*” (FG2 P9). Taken together, these findings demonstrate that caregiving support should extend beyond practical training alone and include emotional validation, psychological support, opportunities for social connection, and interventions aimed at preserving caregivers’ sense of identity and well-being. In summary, caregivers portray both loneliness and self-care as lived experiences marked by suffering, time constraints, and stress; professionals focus on practical strategies to foster community connections and regard self-care as an essential responsibility of the caregiving role; meanwhile, academics analysed the broader effects of isolation on family social structures and conceptualize self-care as a psychological competence that can be strengthened through digital media.

Overall, emotional burden emerged as a multidimensional construct encompassing fear, uncertainty, guilt, social isolation, and, in the most severe cases, the loss of personal identity. Although caregivers primarily sought emotional validation and practical guidance, healthcare professionals emphasised technical competence and patient safety, whereas academic participants highlighted the potential of immersive technologies to integrate experiential learning with empathy-building strategies.

## 4. IVR Scenarios Developed in Response to Identified Training Needs

Based on the educational needs identified through the focus groups, five IVR scenarios were developed to address caregivers’ expectations and improve both their understanding of stroke-related impairments and their practical caregiving skills.

The definition of IVR scenarios followed a three-step process:

(1) Mapping of thematic categories with highest frequency of mention across focus groups;
(2) Matching each category with concrete caregiving tasks amenable to virtual simulation, based on discussion within the multidisciplinary research team (nursing, nutrition, rehabilitation, IT);
(3) Feasibility assessment by IVR developers regarding technical implementation.

Based on the priorities identified during the stakeholder focus groups, five IVR scenarios were developed and categorized into two primary training modules designed to address knowledge gaps and improve caregiver safety.

### 4.1. Enhancing Understanding of the Patient’s Experience

The first module focuses on fostering empathy by allowing caregivers to experience the patient’s sensory and motor limitations firsthand (Table 2).

**Figure 2:**
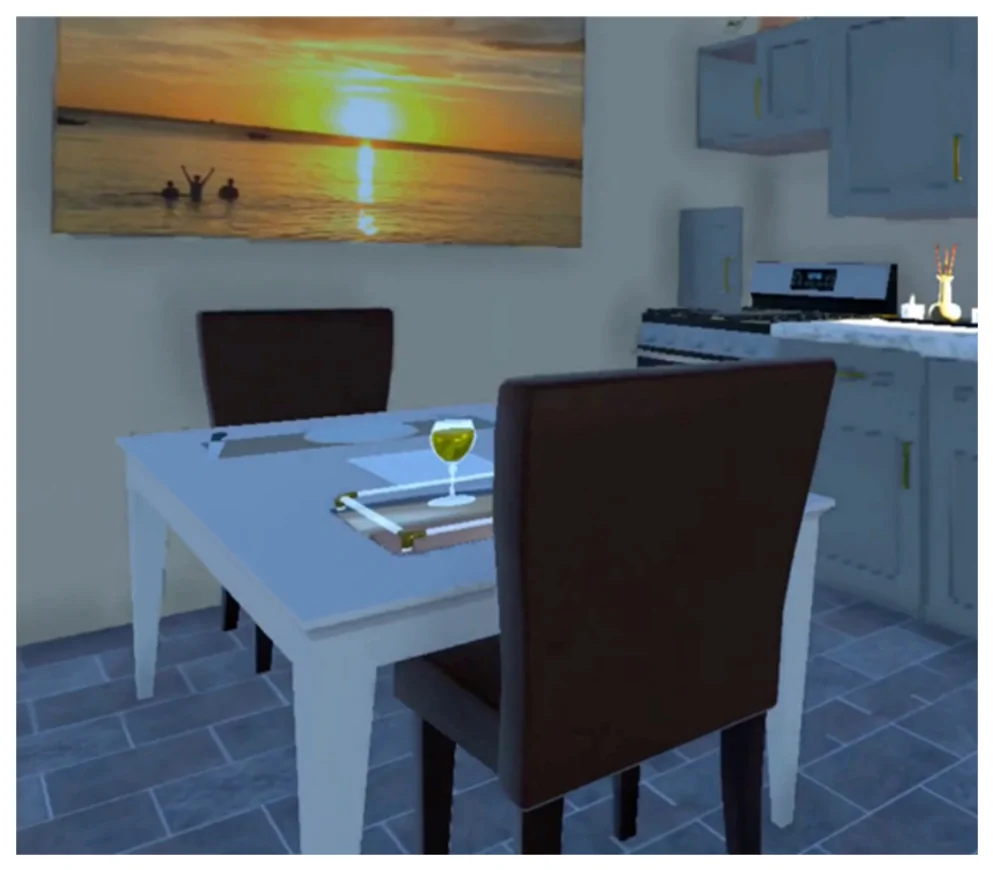
Kitchen scenario of the caregIVR immersive virtual reality environment, designed to simulate motor coordination difficulties and dysphagia-related feeding tasks. Source: CaregIVR project development team (Project: 101129454—caregIVR—EU4H-2022-PJ-3). Reproduced with permission.

**Figure 3:**
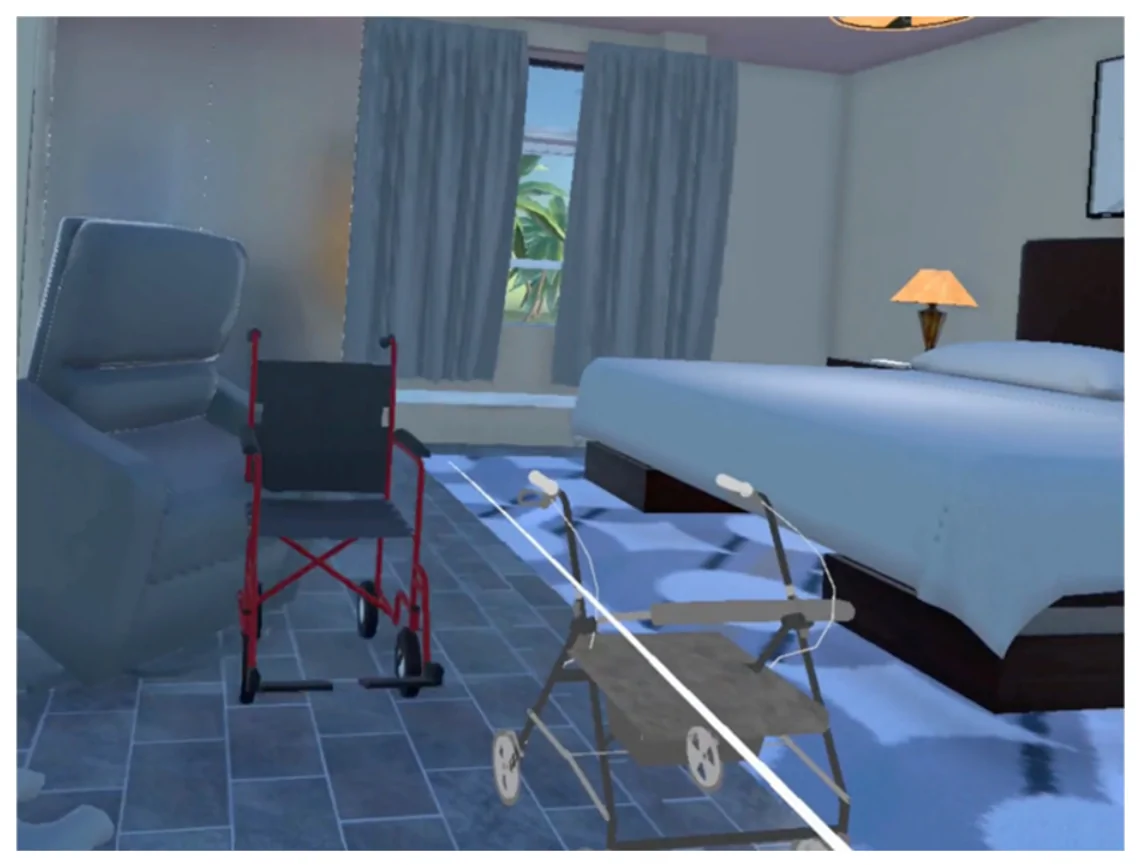
Bedroom scenario of the caregIVR immersive virtual reality environment, designed to train caregivers in patient transfer and bed/bath activities. Source: CaregIVR project development team (Project: 101129454—caregIVR—EU4H-2022-PJ-3). Reproduced with permission.

- Movement Coordination (Kitchen Scenario): In this activity, the participant sits at a kitchen table and attempts to drink a glass of water. The simulation introduces difficulties in controlling and coordinating upper limb movements, potentially causing the participant to spill water, thereby illustrating the frustration of motor deficits (Figure 2).
- Communication (Bedroom Scenario): This scenario simulates aphasia or cognitive communication barriers by placing the participant in a conversation where they cannot understand any words spoken by a family member, perceiving the speech as if it were in a foreign language (Figure 3).

### 4.2. Module 2: Enhancing Practical Caregiving Skills

The second module targets the technical competencies required for safe home-based care in realistic settings (Table 3).

- Feeding and Dysphagia (Kitchen Scenario): Addressing one of the highest-anxiety tasks, this scenario requires participants to select and prepare appropriate food portions for a patient with dysphagia. Caregivers must practice feeding at the correct pace and learn to identify critical warning signs of choking or aspiration (Figure 2).
- Bed/Bath (Bedroom Scenario): This simulation guides the caregiver through a structured sequence of hygiene tasks, including undressing, washing, skin care, changing the patient’s position (decubitus), and changing bed linens while keeping the patient warm (Figure 3).
- Wheelchair–Bed Transfer (Bedroom Scenario): Focused on mobility, this activity trains the caregiver to safely move a patient from a wheelchair to the bed using a transfer board and providing clear instructions to the patient.

Each of these IVR activities concludes with educational feedback, providing specific information that explains the clinical situation and offers practical strategies to help the patient, thereby transforming the training into a repetitive and low-risk learning experience.

While caregivers struggle to interpret the “new” language and behaviours of their family members and actively seek ways to feel more competent and less isolated in managing daily care, professionals emphasize the need for a technical understanding of symptoms, safety, and practical caregiving skills through more extensive training, whereas academics aim to bridge these gaps by developing digital tools that foster artificial empathy, simulate cognitive deficits, and provide personalized, experiential learning opportunities.

## 5. Discussion

The present study provides new insights into the educational and psychosocial needs of informal caregivers during the transition from hospital to home following stroke. The findings confirm that this transition represents a critical period characterised not only by increased caregiving responsibilities but also by uncertainty, emotional distress, and insufficient preparation [3].

Although informal caregivers represent a fundamental pillar of health and social care systems worldwide, inadequate preparation may compromise both caregiver well-being and patient outcomes [34]. Consistent with previous studies, participants identified multidimensional burdens affecting emotional, physical, social, and relational domains [18]. Poor caregiver preparedness has also been associated with lower quality of care, increased risk of adverse events, medication errors, and higher rates of hospital readmission among stroke survivors [15]. Taken together, these findings suggest that caregiver preparedness should be regarded as a core component of stroke rehabilitation rather than as an optional adjunct to patient care.

One of the most significant clinically relevant findings concerned caregivers’ difficulties in recognising and interpreting the cognitive and behavioural consequences of stroke [35]. Unlike visible physical impairments, these “invisible deficits” were easily misunderstood, interpreted as unwillingness, laziness, or lack of motivation, increasing frustration, interpersonal conflict, and emotional strain within the family context [17]. Similar misunderstandings have been described in previous stroke caregiving studies, where reduced awareness of cognitive sequelae negatively affected caregiver coping and patient-caregiver relationships [36].

Another important finding emerging from the focus groups concerns the anxiety generated by uncertainty regarding the “sequence of care actions” required in daily assistance activities, including personal hygiene, positioning of bedridden patients, swallowing management, and safe mobility transfers [37,38]. Caregivers often feared causing harm due to insufficient practical training. These findings suggest that educational interventions should move beyond the mere transmission of information and instead prioritize experiential, context-based learning opportunities that allow caregivers to repeatedly practice complex procedures until they achieve confidence and perceived competence [39].

Traditional discharge education was frequently criticized for being excessively theoretical, fragmented, and concentrated into a single day, with limited opportunities for hands-on training or emotional support [40]. This observation aligns with current evidence indicating that caregiver education is most effective when based on active learning, simulation, and competency-building approaches rather than passive information delivery alone [41].

Consequently, the present findings support the conceptual framework of the caregIVR project, emphasizing that caregiver interventions must simultaneously address technical competencies, emotional preparedness, and empathic understanding. Importantly, the IVR scenarios proposed in the present study were not developed solely on theoretical assumptions but were directly informed by caregivers’ lived experiences and educational priorities identified through the focus groups.

Within this framework, IVR emerges as a promising educational tool. Existing evidence suggests that IVR-based interventions may enhance experiential learning, increase self-efficacy, and improve emotional engagement in healthcare education [22,23,24]. In the context of caregiving, IVR simulations could provide a safe, low-risk environment in which caregivers can practice care procedures before applying them in real-life settings. Specifically, IVR may enhance caregivers’ technical competence by allowing them to rehearse complex care procedures repeatedly in a safe environment [42] while simultaneously fostering empathy through first-person simulations of stroke-related impairments [43]. Immediate educational feedback may further strengthen learning, self-efficacy, and patient safety [41]. This approach directly addresses the practical and emotional knowledge gaps identified by caregivers, healthcare professionals, and academic stakeholders involved in the study.

Attention should also be devoted to the alarming psychological burden described by participants. Several caregivers highlighted severe social isolation, emotional exhaustion, chronic stress, and, in some cases, suicidal ideation. These findings are consistent with literature documenting elevated levels of depression, anxiety, caregiver burnout, and “social frailty” among individuals providing long-term post-stroke care [10]. Importantly, the study identified an additional unmet need that is less frequently emphasized in previous research: the need for role validation. An important contribution of this study is the identification of caregivers’ need for professional validation. Beyond acquiring technical skills, participants expressed the need to receive reassurance that they were providing appropriate care. This dimension has received comparatively limited attention in previous stroke caregiving literature and deserves greater consideration when designing future support interventions [41]. For this reason, the caregIVR platform could integrate not only immersive training scenarios, but also psychosocial support mechanisms, including peer-support groups, opportunities for interaction with healthcare professionals, and channels for emotional counselling. Such components could contribute to reducing loneliness, strengthening resilience, and promoting long-term caregiver engagement.

Finally, although evidence supports an integrated approach to the care of both stroke survivors and their informal caregivers [40,44], with effective communication as the cornerstone of collaborative care [45], participants in this study reported that such an approach remains an aspiration rather than routine practice.

Overall, these findings reinforce the need to move beyond fragmented discharge education towards integrated, person-centred models of caregiver support that combine technical training, emotional support, and innovative digital educational tools.

### Limitations and Future Developments

Despite providing valuable findings, several limitations should be acknowledged.

First, selection bias may have occurred because participants were recruited through associations and institutions already engaged in caregiver support activities. Moreover, the qualitative and descriptive design of the study and the relatively small sample size may limit the transferability of the findings. The results reflect the lived experiences of participants within the Portuguese socio-cultural and healthcare context, which may differ from those observed in other countries and care systems. As the caregIVR project is conceived as a multicentre European initiative aimed at improving health literacy through immersive simulation, an essential next step will involve cross-cultural comparison among the participating countries. Such analyses will be crucial to identify universal caregiver needs that transcend national and cultural boundaries, while also recognizing context-specific differences that may require localized adaptations of IVR interventions.

Second, although the participant sample included individuals aged between 21 and 66 years, older caregivers were underrepresented. Considering that older adults frequently assume caregiving responsibilities after stroke, future studies should ensure greater inclusion of elderly caregivers, particularly given the potential challenges related to digital literacy and the adoption of immersive technologies.

Third, the use of focus groups may have introduced a potential social desirability bias, as participants might have been inclined to present socially acceptable responses in a group setting. Furthermore, participant validation of the translated transcripts was not undertaken, which may have limited the confirmability of the findings.

Finally, some caregivers had previously participated in other activities within the caregIVR project. Although this familiarity may have facilitated discussion, it may also have influenced participants’ expectations and responses.

## 6. Conclusions

By providing an in-depth exploration of caregivers’ educational needs during the transition from hospital to home, this study provides a comprehensive understanding of the educational, practical, and psychosocial needs of informal caregivers supporting stroke survivors at home. Four interrelated domains may guide the development of future caregiver training interventions.

First, caregivers expressed the need to understand the caregiving journey, including the expected care pathway, the progression of recovery, and the changing needs of the person with stroke over time. Second, they highlighted the importance of acquiring knowledge and communication skills to effectively manage neuro-sensory and cognitive deficits, which are often invisible yet profoundly affect daily interactions and caregiving responsibilities. Third, caregivers emphasized the need for practical, context-specific training focused not only on the day-to-day management of care at home but also on problem-solving strategies applicable to real-life situations. Fourth, participants described the substantial psychosocial impact of caregiving, expressing the need for educational interventions that support their physical and psychological well-being, preserve their social relationships, and strengthen their self-efficacy and resilience in order to prevent caregiver burden.

Finally, the use of IVR was recognised as a possible valuable educational resource that can enhance experiential learning by simulating realistic caregiving scenarios. However, the IVR should complement and not replace direct interaction with healthcare professionals and the human support, essential for effective training sessions.

Strengthening caregivers’ knowledge, skills, and self-confidence through structured educational programmes, during which the use of new supportive technologies such as IVR, has the potential to improve patient safety, facilitate continuity of care, and contribute to more sustainable healthcare systems. Nevertheless, caregiver empowerment should be viewed as a continuous, person-centred, and multidisciplinary process that begins before hospital discharge and extends throughout the rehabilitation pathway, rather than as a single educational event. Educational interventions should therefore be tailored to caregivers’ evolving needs and delivered in a supportive, non-judgemental manner, as approaches perceived as overly demanding may inadvertently increase feelings of guilt, inadequacy, and emotional distress.

The findings also underscore the importance of multidisciplinary collaboration involving nurses, rehabilitation professionals, nutrition specialists, physicians, and other healthcare providers in preparing caregivers for the transition to home-based stroke care. Within this framework, nurses are uniquely positioned to coordinate caregiver education and facilitate the integration of innovative simulation-based approaches, such as IVR, with personalised emotional support and practical guidance. Such an approach may enhance caregivers’ preparedness, confidence, and engagement while promoting safer and more effective home care.

## Figures and Tables

**Table 1:** Characterisation of focus group participants by group, sex and age (n = 33).

FG 1—Informal Caregivers	FG 2—Health Professionals and Other Stakeholders	FG 3—Academics and Healthcare Students
P1**—**Female**—**50	P1**—**Female**—**52	P1—Female—27
P2—Female—66	P2—Female—40	P2—Female—23
P3—Female—58	P3—Male—44	P3—Female—21
P4—Female—58	P4—Male—55	P4—Female—21
P5—Male—38	P5—Female—50	P5—Female—41
P6—Female—46	P6—Male—57	P6—Female—34
P7—Male—57	P7—Female—52	P7—Female—55
P8—Female—47	P8—Female—47	P8—Female—36
P9—Female—47	P9—Female—41	P9—Female—39
	P10—Female—54	P10—Female—28
	P11—Female—36	P11—Female—29
		P12—Female—46
		P13—Female—34

Source: Authors’ own elaboration based on focus group data (2024).

**Table 2:** IVR scenarios developed to foster caregivers’ understanding of the patient’s experience.

Activity Name	Understanding the Patient Condition and Limitations–Movement Coordination	Understanding the Patient Condition and Limitations–Communication
Activity description	Participant sitting in front of a table with a glass of water. The participant tries to grab the glass and drink the water but has difficulty controlling and coordinating the movement of his upper limb. When not done properly the participant cannot take the glass to the mouth and may spill some water. At the end of the scene there should be some information explaining the situation and strategies to help the patient.	Living room. Participants sitting or standing, talking about a known subject. The participant doesn’t understand any word that caregiver is saying. It seems like the familiar is talking in another language. At the end of the scene there should be some information explaining the situation and strategies to help the patient.
Scenario	Kitchen	Living room
Scenario description	Kitchen table, patient sitting in front of a table with a glass of water.	Living room. Participants sitting or standing, talking

Source: Authors’ own elaboration within the caregIVR project (2024).

**Table 3:** IVR scenarios developed to improve caregivers’ technical caring skills.

Activity Name	Feeding the Patient-Leading with Dysphagia and Possible Complications	Bed Bath	Wheelchair-Bed Transfer
Activity description	The activity is to feed the patient with dysphagia and identify possible complications. The participant has to select and prepare the food from different given options. After has to select the right portion amount and gently feed the patient. The participant then has to repeat this task giving the correct time for the patient to swallow. It also has to identify signs of choking or aspiration. At the end of the scene there should be some information explaining the situation and strategies to help the patient.	The activity is to give a bed bath. The participant must do the task respecting the different phases, times and order: undress the patient, wash, dry, skin care, change decubitus, wash, dry, skincare, change the bed clothes, keep the patient warm, dress, positioning. At the end of the scene there should be some information explaining the situation and strategies to help the patient.	The activity is to move a patient from the wheelchair to bed. The patient should be instructed how to transfer with a transfer board. At the end of the scene there should be some information explaining the situation and strategies to help the patient.
Scenario	Kitchen table	Bedroom	Outside in a parking lot
Scenario description	Kitchen table, patient sitting on the chair with the participant in front of the patient	Room. Patient on the bed, participant executing the task near the bed	Patient in a wheelchair and the participant nearby giving instructions and helping the patient to transfer

Source: Authors’ own elaboration within the caregIVR project (2024).

## Data Availability

The datasets presented in this article are not readily available because the data are part of an ongoing study. Requests to access the datasets should be directed to the corresponding author.

## Public Involvement Statement

Aim: To directly integrate the lived experiences, priorities, and educational needs into the design and technological development of an Immersive Virtual Reality (IVR) educational tool as part of the European caregIVR project (EU4Health Programme, Grant No. 101129454). Methods: Multilevel engagement of 33 key stakeholders across three complementary focus groups: informal caregivers (n = 9); healthcare professionals and stakeholders (n = 11); academics and health students (n = 13). Study results: PPI led to the classification of 4 core thematic need areas (Caregiver journey, Understanding invisible deficits, Practical adaptation & home care, Psychosocial impact & isolation) and to the co-design IVR Scenarios. Key Outcomes: Inclusion of real-world priorities often omitted in standard clinical training, such as choking anxiety, administrative/legal rights guidance, and caregiver isolation. Discussion and Conclusions: Public involvement fundamentally guided the direction of the study and technical development. The public conceptualized IVR not as a replacement for human touch or healthcare professionals, but as a complementary, risk-free training environment to practice repetitive tasks, build self-efficacy, reduce fear of errors, and reinforce the caregiver’s role within the multidisciplinary care team. Reflections and Critical Perspective: Focus group dynamics may have constrained some individuals from sharing sensitive emotional burdens (Social Desirability Bias).

## Guidelines and Standards Statement

This manuscript was drafted against the COREQ (Consolidated Criteria for Reporting Qualitative Research) guidelines (Tong, A., Sainsbury, P., & Craig, J., 2007. Consolidated criteria for reporting qualitative research (COREQ): a 32-item checklist for interviews and focus groups. International Journal for Quality in Health Care, 19(6), 349–357) for qualitative (focus group) research [26].

## Appendix A. Scheme of Focus Group’s Guide

The guide comprised the following thematic sections:

− Participation motivation. This section explored the reasons that motivated participants to take part in the focus groups (e.g., What motivated you to participate in this focus group?).
− Informal caregivers’ training needs related to patient care. This section aimed to identify caregivers’ knowledge gaps, educational needs, and support requirements in order to inform the development of targeted training interventions to improve the quality of home-based stroke care. Participants were asked about the main challenges encountered in their caregiving role, the information they considered most useful in their daily practice (e.g., patient health management, caregiving activities, nutrition, physical activity, sleep, available community services, social support, financial and legal assistance), and the type of guidance they expected to receive.
− Informal caregivers’ mental health and well-being. This section explored caregivers’ awareness of the emotional and psychological burden associated with caregiving, its impact on their daily lives, and the types of support that could promote their well-being. Questions addressed the areas of life most affected by the caregiving role, the need for information or interventions aimed at preserving emotional well-being, and the self-care domains considered most important.
− Technological tools (interactive voice response [IVR] system, mobile application, and website). This section investigated participants’ expectations regarding the use of digital technologies as educational and support tools. Caregivers were asked to identify the medical or functional aspects of stroke care they found most difficult to understand, the type of ongoing support they would expect from these technologies, and the outcomes or indicators they would consider relevant for evaluating their effectiveness.
− Closing reflections. The final section provided participants with the opportunity to share additional comments and summarize the main topics discussed during the focus group (e.g., Is there anything else you would like to add? Would anyone like to summarize the key points discussed during this session?).
